# Supplement use in sport: is there a potentially dangerous incongruence between rationale and practice?

**DOI:** 10.1186/1745-6673-2-4

**Published:** 2007-05-29

**Authors:** Andrea Petróczi, Declan P Naughton

**Affiliations:** 1 School of Life Sciences, Kingston University, Penrhyn Road, Kingston upon Thames, Surrey KT1 2EE, UK

## Abstract

**Background:**

Supplement use by athletes is complex and research supports the alarming notion of misinformed decisions regarding supplements.

**Hypothesis:**

A frequent divergence between the type of supplements chosen by athletes and the rationale dictating the supplement use is hypothesized. Thus, a potentially dangerous incongruence may exist between rationale and practice.

**Testing the hypothesis:**

In the continued absence of reliable data on supplement use, an alternative approach of studying the reasons underlying supplement use in athletes is proposed to determine whether there is an incongruence between rationale and practice. Existing data from large scale national surveys can be used to investigate this incongruence.

**Implications of the hypothesis:**

In this report, analyses of distinctive patterns between the use and rationale for use of supplements among athletes are recommended to explore this potentially dangerous phenomenon.

## Background

'Supplement' is an overarching name for vitamins, minerals, herbal remedies, traditional Asian remedies, amino acids and other substances to be taken orally. They may also be referred to as dietary, food or nutritional supplements or ergogenic aids (supplements purported to improve athletic performance) and are typically sold in the form of tablets, capsules, soft gels, liquids, powders, and bars. In the UK, most supplements are regulated as foods and subject to the general provisions of the Food Safety Act 1990, the Food Labelling Regulations 1996 and the Trade Descriptions Act 1968. Supplements are not required to exhibit efficacy before marketing, nor are they subject to prior approval unless they are genetically modified or claimed to be new. Medicinal claims on packaging or in an advertisement for a supplement, however, are prohibited.

Widespread debate has accompanied the introduction of new legislation on the use of dietary supplements within the EU. Comprehension of detailed studies, ranging from quantities and patterns of use to side-effects of supplement consumption, has been impeded by variations in terminology and practice amongst countries and user groups. Some thirty thousand supplements are commercially-available in the USA [1] with approximately half of the adult female population being regular users [2-4], with possible adverse effects of unregulated supplement use on health and disease outcomes being of particular interest [1].

## Supplement use in sport

For some 50 years, competitive sports have operated under strict regulation, and adherence to the ever-growing list of prohibited substances [5] is expected from all high performing athletes at all times. Gaining competitive advantage, however, is more important than ever. Personal satisfaction as well as the athletes' livelihoods and their organisations' prosperity depend on success. Athletes naturally turn to supplements hoping to find herbs, vitamins or minerals that provide the desired competitive edge.

Worldwide supplement use among athletes, on average, ranges between 40 and 60 percent [6-12]. Nutritional supplements are typically used for their actual or anecdotal physiological effects in increasing performance and endurance, health maintenance or preventing injuries [13-16], and the extent and amount of ergogenic 'drugs' and supplements used by athletes shows a growing trend [17]. Research linking supplement use to involvement in physical activity and previous studies on decision making patterns among these groups has focused on user subgroup classifications [18-23]. While this enables an understanding of the gross difference in the patterns of use between groups of users, it fails to give an explanation for why those differences might occur. One key understudied aspect is a potential mismatch between the decision making and execution in practice.

Numerous factors can be involved in athletes' decisions to use supplements including desired end points such as increasing strength, endurance, training duration and overcoming injury as well as avoiding sickness and compensating for poor diet. Unfortunately, lack of knowledge and/or misconceptions regarding supplements within athlete populations have been documented for more than a decade [21,24-27]. Recent research also shows that athletes are willing to take supplements based on personal recommendation without gathering reliable information about the substances, often obtaining them directly from retailers and internet sites [28,29]. Adolescents are more willing to take supplements obediently if they are informed by their parents/guardians, as opposed to by coaches or resulting from published research [30].

Conflicting reports on knowledge levels within health care professions demonstrate a wide variation in practice. In one study, physicians and medical students were tested to determine the level of their knowledge regarding efficacy and toxicity, and drug interactions with herbal remedies, and it was found that the mean test scores were only slightly higher than scores obtained from random guessing [31]. On the contrary, recent research among physicians, nurses, nutritionists and pharmacists showed adequate knowledge (average 66% on the knowledge test), less confidence (55%) but noted a serious lack of communication skills (average 2.2 out of 10) regarding herbs and nutritional supplements [32]. Athletic trainers and coaches were found to be reasonably knowledgeable, especially those working with female athletes and/or having more than 15 years of experience [33].

## Supplement types and undesirable consequences

A central issue in researching supplement use is the paucity of regulatory control of supplement providers coupled to a poor understanding within the user community. However, in broad terms many supplements have been associated, rightly or wrongly, with performance enhancement and/or health maintenance [10,15,34-37] including: caffeine, ephedrine, creatine, whey protein, antioxidants, ginseng, multivitamins, vitamin C, iron, Echinacea and magnesium supplements. To illustrate the complexities of studying this field, some thirty thousand individual commercially-available supplements exist [1] and over 60 supplements were listed in a recent UK survey [38], summarised in Table 1.

**Table 1:** Supplements taken by high performance UK athletes (in alphabetical order)

**Listed by product/brand names**	**Listed by components/contents**
Ache Free, Cyclone, Build Up, Green Magic, Herbalife, Hydroxycut, Immune Support, Kalms, Lactibiane, Leppin, Lucozade, Met-Rx, Minadex, Mega EPA, MSN, Multibionta, Musashi Protein, Qlo, Slim Fast, SportsFlex, Vitabalance	Aloe Vera, Alpha-lipoic Acid, Amino Acids, Arnica, Black strap molasses, Calcium, Calendula, Carbohydrate & recovery drinks, C-Glutamine, Chinese Tea, Chromium, Chondroitin, CLA (conjugated linoleic acid), Cod liver oil, Coenzyme Q10, Colostrum, Cranberry juice tablets, Digestive enzymes, Dried skimmed milk powder, Echinacea tea bags, Electrolytes, Evening primrose oil, Ferrous gluconate, Fish oils, Flax seed oil, Folic acid, Garlic capsules, Glucosomine, Harpagophytum procumbens, Hydroxybetamethylbutyrate, L-Carnitine, L-Glutamine, Maitake mushroom, Matltodextrin/Aspartame, meal replacement, Multi Mineral Supplements, Olbas Oil, Protein drinks, Selenium, Soya protein, Starflower oil, Sumpast, Tribulous Terrestris, Vitamin B, B combined with Forceval capsules, Vitamin B complex, Vitamin D and E, Zinc

Beyond contaminated products that easily lead to adverse results in doping tests [8,39-45], vitamin products with accurately listed compounds and substances can also be harmful. High levels of vitamin and mineral intake can lead to toxic side effects [10,35]. For example, the use of iron supplementation by elite athletes is not uncommon and whilst iron is beneficial for athletes with iron deficiency, it can also cause harm with long-term use [46] or certain medical conditions [47]. Similarly, excess intake of vitamin C [48] can be harmful as well as in combination with iron, which may cause damage to the gastrointestinal tract (GI) and initiate or aggravate symptoms associated with chronic GI disorders [49]. The long-term effects of creatine are still unknown but short term side-effects such as cramping and dehydration have been reported along with the suggestion for its use to be under medical supervision [50]. Caffeine is no longer on the list of the IOC's prohibited substances [53]. However, as athletes can use it in training and competition, the relationship between caffeine intake and resulting side-effects such as high blood pressure warrant further study [52,53]. Whilst the controversial natural stimulant, ephedrine, has a threshold (concentration in the urine exceeds 10 μg/ml) for consideration for doping [51], the serious harm, which may be caused by ephedrine is well documented [53-55] and the direct evidence eventually led to a ban on ephedrine in 2004 by Food and Drug Administration (FDA), USA. While the use of blood doping and erythropoietin (EPO) are prohibited, cobalt is not included in the World Anti Doping Association's list of prohibited substances [5]. Cobalt produces similar effects to hypoxia and results in enhanced erythropoiesis, thus in improved sport performance but such practice may be harmful [56].

The aim of this report is to highlight the difficulties involved in generating accurate data on supplement use and abuse in the absence of rigorous regulatory control. A new approach is proposed to break through the conundrum presented by this 'catch 22' situation. Thus, establishing motive-use relationships for supplements, should inform more beneficial use including the elimination of adverse side-effects.

A frequent divergence between the type of supplements chosen by athletes and the rationale dictating the supplement use is hypothesized. Thus, a potentially dangerous incongruence may exist between rationale and practice.

## Testing the hypothesis

Congruence between rationale and supplements used by athletes can be investigated by analyzing quantitative or qualitative data. Although qualitative approaches have the advantage of producing deep understanding of athletes' motivations, data from these studies are not suitable for hypothesis testing. Results from quantitative analyses, assuming adequate sampling and appropriate statistical analyses are objective and comparable. Data from this type of research are normally collected via surveys or structured interviews. Due to the ever present probability of response bias, the use of an anonymous questionnaire is preferable over face to face interviews [57]. The fact that socially desirable responding increases as the sensitivity of the issue being investigated increases [58] should also be taken into consideration.

A sample is recommended to be drawn from a wide cross-section of the athlete population with adequate data in each subgroup of interest. The minimum required sample size depends on the effect size and power of the test [59] but usually is in the magnitude of hundreds in total with a minimum of 20–25 per subgroup in case of continuous variables [60] and a minimum of 5 observed cell frequencies in cross classified categorical variables [61].

Empirical data collection regarding supplement use among athletes can be prospective or retrospective. Retrospective studies collect information about the past and relies on the recollection of events whilst other studies investigate the present or follow participants over the period of time in longitudinal studies. A longitudinal research design is useful when a change over time is important. This would be especially relevant in research aimed to investigate decision making patterns and influences regarding the use of ergogenic aids. Alternatively, one may attempt to derive the same results from a cross-sectional design. However, results are only valuable if data obtained from different age groups adequately represent natural progression over time, thus employing the appropriate sampling method is critical.

Prospective studies have the advantage of using tailored research design and methods with the specific research question in mind. The level of measurement needed for specific analytical procedures and the statistical power-effect size-sample size triangle (if known) can also be given *a priori *consideration. Published studies may be used as pilot studies for estimating effect size and power, hence determining the minimum sample size required for noteworthy research.

In parallel to the increase in computational power, an increasing number of studies utilise existing data that might or might not be collected for the specific research purpose. The least uncomplicated method is re-analyzing existing data to test a new set of hypotheses. At the other end of the spectrum, data mining allows researchers to derive important and previously undiscovered but potentially useful information from large volumes of data using automated searching for meaningful and significant patterns. Prime examples for these types of studies are research projects relying on integrated clinical databases, large and cyclical national or international surveys or centrally held statistical information on populations. Contrary to data mining that requires raw data, meta analysis combines the results of published studies that address the same research question. Although meta analysis is widely used in epidemiological and evidence-based medical studies in order to increase statistical power, this approach is problematic in nutritional supplement research due to the lack of sufficient regulations of the supplement market. These problems are well illustrated in recent meta analyses of antioxidant supplement use for cancer prevention and their effects on mortality [62]. Declared interest and publication bias (i.e., non-significant results are seldom published) should also be taken into account when one attempts meta analyses of supplement use, effects and side effects in athletes.

Considering the costs of research and data collection, especially when a large data set is required, multiple uses of data facilitates a better use of public funds, hence it should be encouraged. However, whether it is a hypothesis driven statistical analysis or structured/unstructured data mining, results from subsequent analyses should contribute to the understanding of the phenomenon. In this case, results must add to the body of knowledge regarding supplement use and rationale in elite athletes.

## Proposed approach

Existing data sets that include both 'supplements' and 'rationale/knowledge' variables can be used to obtain evidence regarding athletes' potentially dangerous, incongruent behaviour. Rationale or knowledge can also be replaced by behavioural intention (if reasons for use are specified) or beliefs about particular substances. Practically, any data that allow a scientifically and statistically meaningful contingency table to be formed are suitable for such analyses. It is suggested that existing, recent, large-scale national surveys conducted among adult and adolescent athletes by national level sport organisations and governing bodies should be scrutinised to test the hypothesis regarding supplement use in sports.

By creating a series of two by two contingency tables from cross-tabling each supplement intake categories and reasons for supplement use, we can:

i) test for relationships between answers (i.e., testing for independence of the two variables);

ii) estimate the strength of this relationship from the proportion of congruent pairs of answers (reasons given for supplement use matches with the reported supplement use);

iii) calculate the relative proportion of answers indicating informed choices and incongruent answers (reasons given for supplement use are not followed by the appropriate supplement) and compare the observed pattern of supplement use to an expected pattern; and

iv) test whether this pattern characterises the athlete population.

A pair of answers is congruent if there is an agreement between an athlete's self-reported supplement use and rationale. The connection is not explicitly made by the athlete but calculated afterwards from answers given on two separate and seemingly independent questions. In surveys of supplement use, athletes are often asked about the substances they have had experience with and some of these surveys also contain explicit questions regarding the reasons behind supplement taking [9,12,21,38,63]. Table 2 illustrates a simplified scenario of one particular reason for supplementing and the corresponding substance. For instance, a group of athletes were asked: i) whether they use supplements to increase their strength and power output; and ii) if they take creatine – a substance known for this effect [10] and currently not sanctioned in competitive sport. Answer options to both questions were limited to dichotomous (Yes/No) responses, thus its contingency table is a two-by-two square. Assume that exactly 100 athletes were asked and 75 (%) of them take supplements to maintain or increase strength. In an ideal case when all athletes make an informed and rational decision about supplements, we would expect *X *to be 75 and *W *to be 25, whereas both *Y *and *Z *should be zero. This {75, 0, 25, 0} is the expected pattern under the assumption of the fully informed choice. On the contrary, a pattern of *X *= 0, *Z *= 75, *Y *= 25 and *W *= 0 would indicate a great deal of confusion or complete misinformation about supplements and their physiological effects.

**Table 2:** Contingency table of creatine use and strength*

		**Reason: increase strength and power output**		
		
		**Yes**	**No**	
**Supplement: creatine**	**Yes**	X	Y	ΣXY
	**No**	Z	W	ΣZW
		ΣXZ	ΣYW	

* ΣXZ = all athletes who use supplements to increase strength, ΣYW = all athletes who does not indicate increased strength as reason for supplement use, ΣXY = all athletes who take creatine and ΣZW = all athletes who do not take creatine.

However, in real life it is not likely that we can observe a perfect pattern, thus we use appropriate statistical analyses [60] to determine whether the observed pattern significantly differs from: i) what is expected; or ii) what would happen by random chance if we assume that the two questions are unrelated. Descriptive statistics obtained from the sample are very interesting and informative but no inferences can be made to the population from which the sample was drawn.

For example, we assume that protein use is common among professional male players from sports where strength is important. In the UK Sport survey [38] 186 male professional players indicated supplement use, of which 106 (57%) listed and 87 (47%) used whey protein. Simple descriptive statistics (frequency and percentage by variables) do not tell us whether the group of 106 players wishing to increase strength contained all 87 who reported the use of whey protein. Therefore, using chi-square tests of association and phi coefficients offer a better approach to delineate athletes' informed choices by considering the two variables (reason for use and supplement used) simultaneously. In this example, the test results shows significant (*χ*^2 ^= 44.28, *p *< .001) and relatively strong (*phi *= .49, *p *< .001) associations between whey protein use and the reason 'maintaining strength' in athletes' answers suggesting that athletes make informed choice regarding strength and protein use. Re-creating the contingency table given in Table 2, shows that *X *= 72 (68%), *W *= 65 (81%), *Y *= 15 (19%) and *Z *= 34 (32%), where ΣXZ and ΣWY are 100%. Comparing these cells to the expected pattern under the assumption of the fully informed choice {106,0,80,0}, we see that: i) 68 % those who wish to maintain strength are taking whey protein and the observed pattern differs significantly from the expected pattern of the fully informed choice (*χ*^2 ^= 10.9, *p *= .001); and ii) 81% of those who are not interested in maintaining strength are not taking protein either and it confirms to the expected pattern (*χ*^2 ^= 2.81, *p *= .094).

The result from the above example is not surprising as protein is a fairly well-known and widely used supplement but the analyses of other substances may not show the same level of confidence. Statistical procedures proposed here (i.e., tests of independence and strength of association) provide evidence that the phenomenon directly evidenced in the sample is observable in the population as well. Because surveys are normally not limited to a sole reason and a single substance, such an approach also allows comparison of strength of associations across supplement categories and reasons.

## Implications of the hypothesis

In order to regulate the European market, the European Union (EU) issued the Food Supplements Directive 2002/46/EC, which was implemented in the UK in 2003, effective from August 2005 [64]. The Food Standards Agency has successfully rebutted the EU's attempt and, by virtue of the derogation in Article 4.6. of the Directive, which permits the continued use of vitamins and minerals not on the 'positive lists', the UK supplement market will remain semi-regulated at least until 2009 [65]. Unless clear evidence is found for adverse effects, health warnings are therefore not likely to be placed on nutritional supplements [9]. Thus, widespread supplement use is likely to remain at a high level or to increase further. The conundrum is obtaining the strong evidence in the absence of regulation which severely limits the validity of clinical investigations.

## Competing interests

The author(s) declare that they have no competing interests.

## Authors' contributions

AP studied published surveys (e.g., the UK Sport survey), conceived the study and performed the statistical analyses for the example. DN added considerations of supplement use and helped to draft the manuscript. All authors read and approved the manuscript.
